# Metabolic Responses in Endothelial Cells Following Exposure to Ketone Bodies

**DOI:** 10.3390/nu10020250

**Published:** 2018-02-22

**Authors:** Erika Meroni, Nadia Papini, Franca Criscuoli, Maria C. Casiraghi, Luca Massaccesi, Nicoletta Basilico, Daniela Erba

**Affiliations:** 1 Human Nutrition Unit, Department of Food, Environmental and Nutritional Sciences DeFENS, Università degli Studi di Milano, Via Celoria, 2, 20133 Milan, Italy; erika.meroni@unimi.it (E.M.); franca.criscuoli@unimi.it (F.C.); maria.casiraghi@unimi.it (M.C.C.); 2 Department of Medical Biotechnology and Translational Medicine, L.I.T.A., Università degli Studi di Milano, Via F.lli Cervi, 93, 20090 Segrate, Milan, Italy; nadia.papini@unimi.it; 3 Department of Biomedical, Surgical and Dental Sciences, Università degli Studi di Milano, Via Saldini, 50, 20133 Milan, Italy; luca.massaccesi@unimi.it; 4 Department of Biomedical Sciences, Surgical and Dental Sciences, Università degli Studi di Milano, Via C. Pascal, 36, 20133 Milan, Italy; nicoletta.basilico@unimi.it

**Keywords:** ketone bodies, ketogenic diet, HMEC-1 cells, oxidative stress, Nrf2 pathway

## Abstract

The ketogenic diet (KD) is a high-fat, low-carbohydrate diet based on the induction of the synthesis of ketone bodies (KB). Despite its widespread use, the systemic impact of KD is not completely understood. The purpose of this study was to evaluate the effects of physiological levels of KB on HMEC-1 endothelial cells. To this aim, DNA oxidative damage and the activation of Nrf2, a known transcriptional factor involved in cell responses to oxidative stress, were assessed. The exposure of cells to KB exerted a moderate genotoxic effect, measured by a significant increase in DNA oxidative damage. However, cells pre-treated with KB for 48 h and subjected to a secondary oxidative insult (H_2_O_2_), significantly decreased DNA damage compared to control oxidized cells. This protection occurred by the activation of Nrf2 pathway. In KB-treated cells, we found increased levels of Nrf2 in nuclear extracts and higher gene expression of HO-1, a target gene of Nrf2, compared to control cells. These results suggest that KB, by inducing moderate oxidative stress, activate the transcription factor Nrf2, which induces the transcription of target genes involved in the cellular antioxidant defense system.

## 1. Introduction

One of the dietary approaches that has attracted particular success in recent years is the “ketogenic diet” (KD). This is a dietary program that was designed in the 1920s as a therapy for drug-resistant epilepsy [1,2], which then gained popularity in the 1970s as a weight-loss diet (Atkins) [3,4]. Currently, there are many diets that are based on this model. Recently, KD has also been proposed for a number of neurological disorders like Parkinson’s and Alzheimer’s diseases [5,6,7].

The KD is a high-fat, low carbohydrates diet planned to achieve a metabolic state called ketosis, characterized by increased levels of circulating ketone bodies (KB), i.e., the compounds acetoacetate (AA), β-hydroxybutyrate (βHB) and acetone. Under normal conditions and with a balanced diet, KB are produced in small quantities, but, under a KD, their synthesis is induced as a result of a very low carbohydrate intake (VLCKD) and high fat intake. The low glucose supply stimulates the catabolism of fats to obtain energy, leading to the accumulation of acetyl-CoA and synthesis of KB by the liver, which are sent to the peripheral tissues where they are oxidized to produce energy [8,9,10].

Despite its multiple applications, the impact of KD on the human body is not completely understood. Several studies have been performed to clarify the mechanisms by which KD achieves neuroprotection [11,12,13], whereas less is known about the systemic effects of KB circulating in the bloodstream. The adverse effects of KD on the cardiovascular system have not been well documented and are still controversial. For instance, some studies observed, in epileptic patients treated with KD, an increase of serum levels of cholesterol, triglycerides and LDL, known risk factors for CVD [14,15], and changes in vascular structure and function are also reported [16]. On the other hand, the consumption of KD, in non-epileptic subjects, has been mostly associated with an improvement in some cardiovascular risk factors, even though the effect of long term use of KD and its lipid composition should be better assessed [17].

Endothelial dysfunction is another key risk factor for CVD, often resulting from cellular oxidative unbalance [18,19]. Consequently, it appears critical to investigate the KB effects on endothelial model, taking into consideration that KB exposure has been shown to alter red-ox cellular state. In hippocampal cells of mice, KB and in particular AA, seem to exert a protective effect, by defending cells against oxidative stress induced by glutamate [11]. Jarrett and colleagues showed that KD stimulates the biosynthesis of reduced glutathione (GSH) in rat hippocampal mitochondria, specifically by increasing mitochondrial GSH levels and improving redox status, thereby resulting in lower production of ROS [12]. A recent review suggests a further potential mechanism of KD, by means of the production of redox signalling molecules. Under these conditions, KD induced adaptive cellular responses in the hippocampus of rats, such as activation of the transcription factor Nrf2 (nuclear factor erythroid 2-related factor 2) [13].

Nrf2 is normally sequestered in the cytoplasm by binding to the protein Keap1 (kelch ECH associating protein 1), an adaptor subunit of Cullin 3-based E3 ubiquitin ligase, which constantly ubiquitinates Nrf2, targeting it for the proteasome where is degraded. When stress occurs, Keap1 is inactivated and Nrf2 is stabilized; however, Nrf2 does not dissociate from inactivated Keap1, so de novo synthesized Nrf2 translocates into the nucleus. There, it forms a heterodimer with small Maf (sMaf) proteins and activates the transcription of target genes by binding to antioxidant response elements (ARE) [20,21,22,23,24,25,26]. 

Among the enzymes up-regulated by Nrf2, one of the most important is HO-1, which catalyzes the conversion of heme into biliverdin, carbon monoxide and free iron, and has both anti-inflammatory and antioxidant properties [27,28,29,30]. It has been seen, in an animal model, that HO-1 increases in the liver of KD-fed rats compared to control rats after three weeks [31]. So, the activation of Nrf2 may be one of the main mechanisms explaining the positive effects exerted by KB [13,31].

In the present study we focused our attention to assess, by in vitro model, the vascular risk represented by KD-induced oxidative stress. We hypothesized that treatment with the KB would induce Nrf2 nuclear localization and antioxidant protein activation, protecting cells against a following oxidative challenge. To this aim we investigated, by an in vitro model of endothelium, (1) whether the exposure to KB might directly exert an oxidative damage to DNA; (2) the ability of KB to modulate DNA susceptibility toward a secondary oxidative insult and (3) the endothelial cell adaptive metabolic response to KB exposure, by activation of the Nrf2 pathway.

## 2. Materials and Methods

### 2.1. Materials

A long-term cell line of human microvascular endothelial cells (HMEC-1) immortalized by simian-virus-40 large T antigen [32] was provided by the Centers for Disease Control and Prevention, Atlanta, GA, USA. Due to its immortalization, it is necessary to consider that this cell line might have acquired some tumor features. However, HMEC-1 has widely been used as a model for studying endothelial dysfunction [33,34,35]. It has been reported that this cell line retains the morphologic, phenotypic and functional characteristics of normal human microvascular endothelial cells [32]. Moreover, comparing different endothelial cell lines, HMEC-1 is considered the most suitable model to investigate microvascular endothelium [36]. All cell culture reagents, chemicals and both β-hydroxybutyric acid (298360) and acetoacetic acid lithium (A8509) were purchased from Sigma Chemical Co. (St. Louis, MO, USA).

### 2.2. Cell Culture

HMEC-1 cells were cultivated in MCDB131 medium supplemented with 10% fetal bovine serum, 10 ng/mL epidermal growth factor, 1 μg/mL hydrocortisone, 2 mM glutamine, 100 U/mL penicillin, 100 μg/mL streptomycin and 20 mM HEPES buffer. Cells were passaged every 3–4 days.

For the analysis, cells were seeded at a density of 5 × 10^4^ cells/cm^2^ in 60 mm Petri dishes and allowed to reach confluence in 24 h; then, cells were supplemented with KB dispersed in complete medium, and incubated at 37 °C in an atmosphere containing 5% of CO_2_ (2–48 h time points), according to the experimental design.

### 2.3. Treatment with AA and βHB

Stock solutions for AA and βHB were prepared in sterilized water. HMEC-1 cells were supplemented with different concentrations of KB: from 2 to 20 mM for βHB and from 0.5 to 5 mM for AA. Previous studies have reported that βHB and AA occur in vivo after the induction of ketosis in a ratio of 4:1, respectively [8]. To simulate the physiological condition both βHB and AA were incorporated simultaneously in cell treatments.

### 2.4. Cell Viability 

Cell viability was determined by the MTT reduction assay [37]. HMEC-1 cells were incubated for 24, 48 and 72 h in 24-well plates after the supplementation with KB. Complete medium containing 10% MTT was added to treated and control cells in each well. The cells were incubated at 37 °C for 2 h, the supernatants were removed, and 1 mL of a solution of isopropanol/1 N HCl (24/1 *v*/*v*) was added. Absorbance was recorded at a wavelength of 590 nm and a reference wavelength of 630 nm using a microplate reader (Multiskan Go, Thermo Fisher Scientific, Waltham, MA, USA). Viability was expressed as the percentage of treated vs. control set at 100%.

### 2.5. Comet Assay

Based on the results of the viability test, cells were exposed to KB (βHB 4 mM, AA 1 mM) for 2, 24 and 48 h at 37 °C with 95% humidity and 5% CO_2_. Every treatment was performed in triplicate; negative (cells without KB) and positive (cells treated with H_2_O_2_: 50 μM for 5 min) controls were included in each batch. After incubation, an aliquot of cells was used to verify cell viability via the trypan blue exclusion test [38], to be sure that cells were suitable for the comet assay. Another aliquot of cells was centrifuged (11,000× *g*, for 15 s; SL16R Thermo Fisher Scientific), re-suspended in 1.5% low melting point agarose, and spread on a microscope slide previously covered with 1% normal melting point agarose. Embedded cells were lysed, DNA was allowed to unwind in electrophoresis buffer (pH 10) and then electrophoresis was performed at 25 V and 300 mA for 20 min. The slides were then immersed in neutralization buffer for 15 min, stained with ethidium bromide and analyzed using a fluorescence microscope (BX60 Olympus, Tokyo, Japan) equipped with Image-Pro Plus software (Immagini & Computer, Bareggio; Milano, Italy). Fifty images were analyzed for each slide and the tail moment was registered. DNA damage was expressed as the percentage of DNA in the tail [39,40].

### 2.6. RNA Extraction and Gene Expression 

Total RNA was isolated with the RNeasy Mini Kit (Qiagen, Hilden, Germany), according to the manufacturer’s protocol. Then, 0.8 μg of RNA was reverse-transcribed employing the iScript cDNA Synthesis Kit (Bio-Rad Laboratories, Hercules, CA, USA). Real-Time PCR was performed using an iCycler thermal cycler (Bio-Rad Laboratories) with cDNA corresponding to 10 ng of total RNA as the template. The PCR mixture included 0.2 μM primers and 1X SYBR Green PCR Master Mix (Bio-Rad Laboratories) in a final volume of 20 μL. The primer sequences are provided in Table 1.

Amplification and real-time data acquisition were performed using the followed cycle conditions: initial denaturation at 95 °C for 3 min, followed by 45 cycles of 10 s at 95 °C and 30 s at 58 °C. The fold change in expression of the different genes in treated HMEC-1 cells compared with control cells was normalized to the expression of GAPDH and was calculated by the equation 2-∆∆Ct using iQ5 software version 2.0 (Bio-Rad Laboratories, Hercules, CA, USA). All reactions were performed in triplicate, and the accuracy was monitored by analysis of the PCR product melting curve.

#### 2.6.1. Nuclear Compartment Isolation

After treatment, cells were harvested by scraping into PBS and were then centrifuged at 400× *g* for 10 min at 4 °C. Cell pellets were lysed for 10 min at 4 °C with a buffer containing 10 mM HEPES pH 8.0, 1.5 mM MgCl_2_, 10 mM KCl, 0.5 mM dithiothreitol (DTT), 0.05% Nonidet P-40 (NP-40), 1 mM Na_3_VO_4_ and protease inhibitors. Lysates were centrifuged at 2500× *g* at 4 °C for 10 min, the supernatants (including cytoplasm) were collected in new tubes and the nuclear pellets were resuspended in a lysis buffer containing 20 mM HEPES pH 8.0, 1.5 mM MgCl_2_, 420 mM NaCl, 1.0 mM DTT, 0.2 mM EDTA, 1 mM Na_3_VO_4_ and protease inhibitors for 30 min at 4 °C. The nuclear extracts were clarified by centrifugation (10,000× *g* at 4 °C for 5 min) and collected in new tubes.

The concentration of protein in the samples was measured by Bradford’s method [41].

#### 2.6.2. Western Blot Analysis

Proteins were denatured by boiling for 5 min in sodium dodecylsulfate (SDS) sample buffer, loaded into 10% SDS-PAGE gels and subsequently transferred onto PVDF membranes by electroblotting. Then, the PVDF membranes were incubated in Tris-buffered Saline with 0.1% (*v*/*v*) Tween 20 (TBS-T) containing 5% (*w*/*v*) non-fat dried milk or 5% (*w*/*v*) bovine serum albumin (BSA; Sigma) for 1 h. Blots were incubated with primary antibodies in the appropriate blocking solution at 4 °C overnight. The following primary antibodies were used: Nrf2 (C-20, Santa Cruz Biotechnology, Inc., Dallas, TX, USA) dilution 1:400 in TBS-T+5% (*w*/*v*) non-fat dried milk; GAPDH (FL-335, Santa Cruz Biotechnology, Inc.) dilution 1:500 in TBS-T+5% (*w*/*v*) non-fat dried milk; lamin A/C (N-18, Santa Cruz Biotechnology, Inc.) dilution 1:500 in TBS-T+5% BSA. Membranes were washed three times for 10 min and then incubated with the appropriate secondary antibody conjugated with horseradish peroxidase for 1 h. For the immunological detection of proteins, the enhanced chemiluminescence system (Pierce Biotechnology, Waltham, MA, USA) was used. The acquisition of PVDF membrane images and the densitometric analysis of blots was performed using an Alliance MINI HD9 (UVItec) apparatus (Cleaver Scientific, Warwickshire, United Kingdom) and related software.

### 2.7. Statistical Analysis

Experimental results re expressed as mean ± SD of three (four for western blot) independent measurements. The data for various parameters were analyzed by one-way analysis of variance (ANOVA) with SPSS Statistics 22 (IBM). Significant differences (*p* < 0.001 for comet assay analysis, *p* < 0.05 for viability, western blot and PCR analysis) were detected by the Tukey test.

## 3. Results

### 3.1. Cell Viability

**Figure 1:**
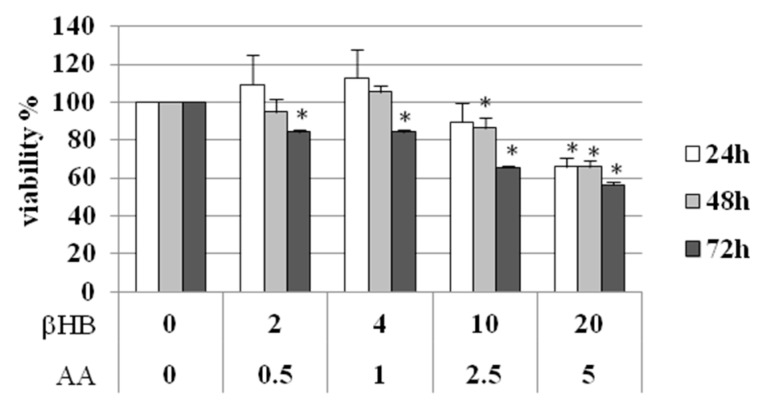
Viability of HMEC-1 treated with KB (βHB 2–20 mM and AA 0.5–5 mM). Data are expressed as mean ± SD. * *p* < 0.05 compared with control (βHB = 0 and AA = 0).

To test the toxicity of KB on endothelial cells, HMEC-1 cells were treated with various concentrations of βHB and AA for different times (24, 48 and 72 h). Concentrations up to 4 mM βHB and 1 mM AA showed no significant effects on cell viability up to 48 h (Figure 1). Higher concentrations were harmful at 48 h, and lower concentrations were damaging at 72 h. Considering this outcome, and assuming that 48 h is an appropriate time to detect a metabolic cellular response to an external stimulus, all further investigations were performed on HMEC-1 cells exposed to 4 mM βHB and 1 mM AA.

### 3.2. Genotoxicity of KB

**Figure 2:**
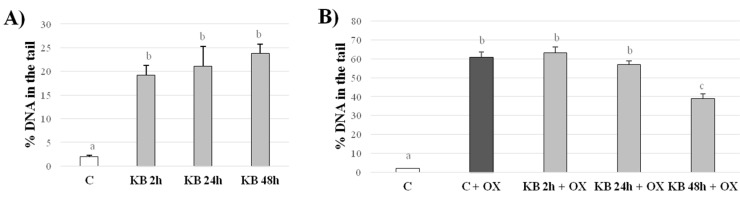
Analysis of DNA damage in HMEC-1 cells treated with KB (4 mM βHB, 1 mM AA) for 2, 24 or 48 h (**A**) and subsequently oxidized with 50 μM H_2_O_2_ for 5 min (**B**); results are expressed as the % of DNA in the tail (mean ± SD). Data not sharing a common letter are significantly different, *p* < 0.001.

DNA damage was measured using the comet assay, which is a method used to quantify the breaking of single strands of DNA (corresponding to oxidized bases) and the formation of alkali labile sites, at the single cell level. This technique has been demonstrated in several studies to have excellent sensitivity in the determination even minimal levels of oxidative damage; moreover, it does not require an excessive number of cells and is simple to perform [39,40]. Figure 2A shows the results relating to DNA damage, both in control cells and cells supplemented with KB (βHB 4 mM and AA 1 mM) for 2, 24 and 48 h. KB induced moderate stress to cells at every incubation time point; in particular, it was found that DNA damage, expressed as the percentage of DNA in the tail, was about to 21% in KB-treated cells vs. 2% in control cells (C = 2.03 ± 0.21, KB2h = 19.25 ± 2.04, KB24h = 21.09 ± 4.20, KB48h = 23.75 ± 2.02% of DNA in the tail, *p* < 0.001). The results show that the DNA damage induction occurred rapidly, but it was not related to the duration of incubation. Nevertheless, KB-mediated oxidative stress was moderate, because the DNA damage was <30%, a minimum level considered by Klinder et al. to be indicative of the genotoxic activity of treatments [42].

#### Effects of KB Exposure on Secondary Oxidative Insult

DNA damage was evaluated in cells supplemented or not with KB (again for 2, 24 or 48 h), and subsequently subjected to an oxidative insult, represented by H_2_O_2_ 50 μmol/L for 5 min. The results are presented in Figure 2B. Firstly, there was a net increase in oxidative DNA damage in control cells exposed to H_2_O_2_ (C = 2.03 ± 0.21 vs. C + OX = 60.92 ± 2.73, % DNA in the tail, *p* < 0.001); cells previously treated with KB and exposed to H_2_O_2_ showed DNA damage as well. However, as the duration of exposure to KB increased, the DNA damage decreased. In fact, there was a significant decrease, equal to −36% (*p* < 0.001), in H_2_O_2_-induced oxidative DNA damage in the cells treated with KB for 48 h (KB48 + OX = 39 ± 2.42% DNA in the tail) compared to the oxidized control cells (C + OX = 60.92 ± 2.73% DNA in the tail). This result suggests a protective effect of KB, which appears able to activate, within 48 h, a positive response by the cells, which confers them protection against a secondary oxidative insult (in this case due to H_2_O_2_).

### 3.3. Gene and Protein Expression of Nrf2

**Figure 3:**
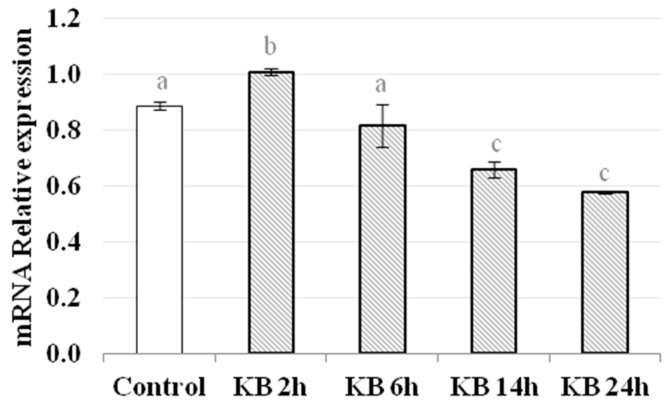
Nrf2 mRNA expression by real-time PCR in control HMEC-1 cells and cells treated with KB for 2, 6, 14 or 24 h. mRNA expression was normalized to the level of the housekeeping gene GAPDH. Data are the means ± SD of three independent experiments. Data not sharing a common letter are significantly different, *p* < 0.05.

**Figure 4:**
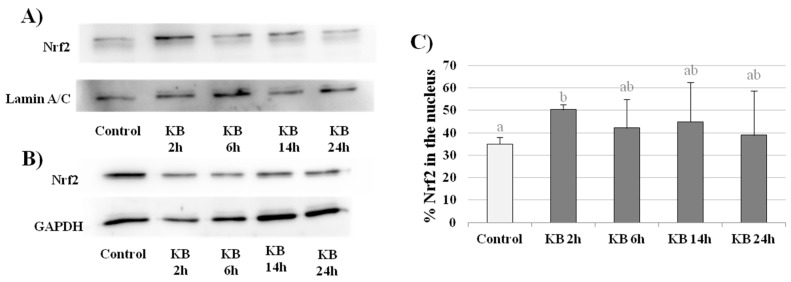
Western blot analysis. Nrf2 protein expression in control cells or cells treated with KB for 2, 6, 14 or 24 h in the nuclear (**A**) and cytoplasmic (**B**) fractions. Lamin A/C was used as a nuclear marker. GAPDH was used as a cytoplasmic marker. The western blot image is representative of four independent experiments (**A**,**B**). Densitometric analysis of Nrf2 protein expression was performed using lamin A/C as the loading control and Nrf2 nuclear translocation are expressed as the % of Nrf2 in the nuclear fraction compared to the total amount of Nrf2 in whole cells. Data are the mean ± SD of four independent experiments. Data not sharing a common letter are significantly different, *p* < 0.05 (**C**).

Nrf2 is a primary transcription factor involved in the cellular response to oxidative stress. When stress occurs, the Nrf2-Keap1 complex is disrupted, resulting in the stabilization of Nrf2, which can translocate to the nucleus where it activates the transcription of target genes by binding to the ARE [20,21,22,23,24]. HMEC-1 cells were exposed to KB for 2, 6, 14 and 24 h. Regarding the gene expression of Nrf2, a significant increase was observed at 2 h in KB-treated cells compared to control cells (C = 0.89 ± 0.02, KB2h = 1.01 ± 0.01, variation about 13% vs. control, *p* < 0.05). On the contrary, at later time points, there was a decrease in Nrf2 mRNA levels (KB6h = 0.82 ± 0.08, KB14h = 0.66 ± 0.03, KB24h 0.58 ± 0.01, *p* < 0.05) (Figure 3). The western blot analysis showed an increase in the total amount of Nrf2 at 2 h (by about 30%) in cells treated with KB compared to the control cells. Moreover, considering only the nuclear extracts, the amount of Nrf2 was greater in KB-treated cells than in the control. Particularly, as reported in Figure 4C, looking at the level of Nrf2 in the nucleus compared to the total amount of Nrf2 in the whole cell (both cytoplasmic and nuclear extracts), there was a significant difference at 2 h in cells treated with KB compared to control cells (KB2h = 50.30 ± 2.2; Control = 34.95 ± 2.9, % vs. total, *p* < 0.05).

### 3.4. Activation of the Nrf2 Pathway: HO-1 Gene Expression

**Figure 5:**
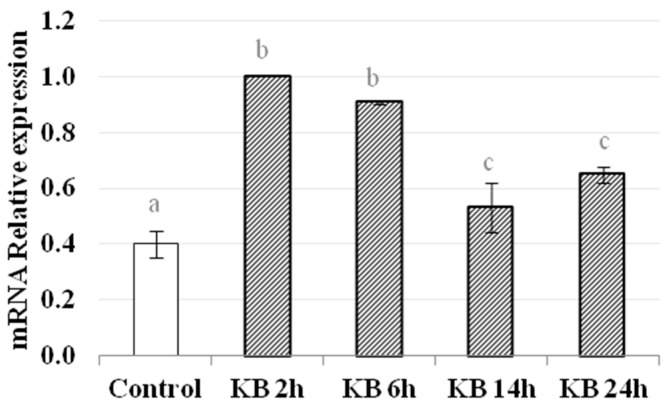
Real time PCR analysis of HO-1 mRNA expression in control HMEC-1 cells and cells treated with KB for 2, 6, 14 or 24 h. mRNA expression was normalized to the level of the housekeeping gene GAPDH. Data are the means ± SD of three independent experiments. Data not sharing a common letter are significantly different, *p* < 0.05.

Activation of the Nrf2 pathway was investigated by assessing the gene expression of HO-1. The data show that HO-1 mRNA levels were significantly higher in cells treated with KB compared to control cells, at all time points measured (Figure 5). The greatest increases in mRNA levels occurred after 2 h and 6 h of treatment with KB (C = 0.40 ± 0.05, KB2h = 1.00, KB6h = 0.91± 0.01, *p* < 0.05), while at later time points there was a decrease, but the values were still higher than in control cells (KB14h = 0.53 ± 0.09, KB24h = 0.65 ± 0.03, *p* < 0.05).

## 4. Discussion

The present study was designed to investigate the effects of exposure to KB in HMEC-1 endothelial cells by measuring markers of oxidative stress and metabolic responses. Primarily, our results demonstrate that βHB and AA, at concentrations equal to 4 mM and 1 mM, respectively, induced moderate oxidative stress, detectable by a significant increase in oxidative damage to DNA after 2 h. 

In literature conflicting data are reported concerning the production of ROS by KB and KD. For instance, in cancer models, some studies [43,44] found ROS reduction owing to KB metabolism, while other studies reported increased oxidative stress [45], or no change [46]. In primary cells, according to Veech, the metabolism of KB should reduce the amount of Q semiquinone, thereby decreasing ROS production in primary cells [47]. However, other investigations showed conflicting results. Shi et al. investigated the pro-oxidant activities of βHB in primary calf liver cells. After treatment with various concentrations of βHB for 24 h, they demonstrated that βHB increased the oxidizing species and decreased antioxidant defenses, causing stress at least within 24 h [48]. Another study, using mitochondria from the hippocampus of rats fed a KD, seem to confirm this thesis, as an increase in H_2_O_2_ was shown at the beginning of the diet, while a decrease was observed after more extended periods of the diet. Moreover, the levels of 4-HNE were also higher in the KD group compared to controls. Overall, these data suggest the induction of mild oxidative stress immediately after the beginning of a KD [31]. Regarding endothelial cells, it has been demonstrated that KB can generate oxygen radical and overall contribute to increased oxidative stress. In particular, it was shown that elevated levels of KB can result in lipid peroxidation, which is apparently increased by oxygen radicals generated by AA [49]. Accordingly, the study of Kanikarla-Marie et al., confirmed these results demonstrating, in HUVEC cells, that ketones cause increased oxidative stress by up-regulating NADPH oxidase 4 [50]. Thus, recent data are in agreement with the results highlighted by our analysis, which underlines the induction of moderate oxidative stress at the cellular level due to the presence of KB. However, this stress was not related to the duration of exposure. We did not find any significant differences in DNA damage among the three time points of exposure (2, 24 and 48 h). It might be hypothesized that, during 48 h of treatment, the oxidative stress generated at 2 h induced some cellular metabolic responses. The activation of antioxidant enzymes could be one of the protective mechanisms promoted by the cell as a consequence of KB-induced stress.

In order to investigate our hypothesis regarding the cellular response to KB, we decided to expose HMEC-1 cells to KB and later to a secondary oxidative insult, in our study represented by H_2_O_2_. Among the stimuli used to produce quantifiable oxidative damage to DNA and modulated by the presence of antioxidants, hydrogen peroxide has proved to be the most effective and reproducible [51,52]; therefore, it was used in this study. Surprisingly, oxidized cells previously treated with KB for 48 h showed significantly less DNA damage compared to control oxidized cells. This interesting result suggests that KB, by causing moderate oxidative stress detectable based on oxidative DNA damage, at a subsequent time point, are able to activate a cellular response that results in protection against a secondary insult. The beneficial effects of KB have been investigated before to understand their role in some pathologies, above all in epilepsy, but also in other neurological diseases and cancer. In particular, Noh et al. discovered that AA protects neuronal cells from oxidative glutamate toxicity. Their study was showed a significant decrease in glutamate-induced ROS production in cells treated with AA compared with cells treated with glutamate alone [11]. To test the possible protective role of βHB against oxidative stress, Shimatzu et al., implanted mice with subcutaneous pump delivering βHB or PBS for 24 h. Then mice received an intravenous injection of paraquat, which produces superoxide anions. The authors found that paraquat treatment of control mice receiving PBS led to an increase in carbonylated proteins, while this increase was significantly prevented in βHB-treated mice. Similarly, the increase of 4-HNE was suppressed in mice treated with βHB [53]. Another interesting paper published by Jarrett and colleagues reported that KD increases mitochondria GSH levels, resulting in decreased mitochondrial ROS production and the protection of mitochondrial DNA in rats fed a KD for three weeks [12]. Consistently with these published studies, our data suggest the capacity of KB to promote cellular responses that result in the prevention of oxidative damage induced by a secondary stressor, possibly by activating the antioxidant defense system.

One possible mechanism by which this protection might occur is through activation of the Nrf2 pathway [13,54]. As a transcription factor, Nrf2 regulates many adaptive cytoprotective responses to counteract the deleterious effect of ROS. Several studies have indicated that increased nuclear translocation of this protein protects against oxidative stress injury [55,56]. Therefore, we first evaluated Nrf2 gene expression, then its translocation into the nucleus. We decided to expose HMEC-1 cells to KB for 2, 6, 14 or 24 h, because we hypothesized that the activation of a cellular response would happen quickly, as we saw protection at 48 h. According to the gene expression data, it seems that there was very rapid stabilization of Nrf2, which implies the fast de novo synthesis of Nrf2, as we saw higher mRNA Nrf2 levels at 2 h. Even though we did not observe higher levels of Nrf2 mRNA expression from 6 to 24 h, this does not mean that Nrf2 was not active. Actually, it has been previously reported that some agents can increase the nuclear translocation of Nrf2, but not alter its gene expression level [55,57,58]. Moreover, these findings on the gene expression of Nrf2 are consistent with the results obtained from the western blot analysis, which showed an increase in the total amount of Nrf2 in KB-treated cells compared to control as well as nuclear accumulation already at 2 h. This trend regarding Nrf2 nuclear accumulation implies that the link between Nrf2 and Keap1 has changed, leading to stabilization of the transcription factor, which was not degraded by the proteasome, thereby promoting its translocation into the nucleus. It could be possible that KB initially causes the production of low levels of ROS, which may serve as a redox signaling stimulus and activate the transcription factor Nrf2. In 2010, Milder et al. proposed for the first time that the consumption of KD activates the Nrf2 pathway. Indeed, they observed Nrf2 accumulation in nuclear fractions from the hippocampus and liver of rats fed a KD for up to three weeks, suggesting chronic Nrf2 activation and nuclear translocation [13]. Most recently, another research group investigated the impact of KD on tumor growth in an animal model, and reported that one of the protective mechanisms against cancer was the stimulation of several factors, including Nrf2 [54]. Although the dietary intervention with KD was different in the two studies, both of them clearly showed higher Nrf2 nuclear levels.

To further confirm the involvement of the Nrf2 pathway, we analyzed the gene expression of HO-1, one of its target genes. Among the antioxidant proteins highly regulated by Nrf2, HO-1 was chosen because the redox-dependent Keap1/Nrf2 system plays a central role in HO-1 induction in response to oxidative stress [29,55]. The results show an increase in HO-1 gene expression at all time points, notably after 2 and 6 h of KB treatment, confirming activation of the Nrf2/HO-1 pathway, in accordance with published studies [55,59,60]. A number of studies have shown that activation of the Nrf2/HO-1 pathway protects different kinds of cells [55,56,57,58] and, additionally, this pathway has been considered in papers specifically related to the effects of KD [13,54]. In particular, Milder et al. proposed a new mechanism of action of KD that involves Nrf2 activation. They suggested that the Nrf2 pathway is systemically activated by KD via redox signalling, leading to chronic cellular adaptation and the induction of protective proteins. Indeed, in their study, both Nrf2 and HO-1 protein expression were higher after the consumption of a KD [13]. Furthermore, a more recent study that investigated the long-term effects of a KD in order to understand its effect on tumor growth confirmed the hypothesis described above. In fact, the authors observed an increase in Nrf2 levels in rats treated with a KD compared to control [54]. Both these studies are in accordance with the results obtained in the current study and overall demonstrate rapid activation of the Nrf2 pathway after KB-induced stress.

Further studies, planned in order to evaluate the effects of AA and βHB individually supplemented, should clarify their specific influence on DNA oxidative damage and Nrf2 activation. Moreover, it will be interesting to assess the expression of multiple Nrf2 target genes, in addition to HO-1, to further confirm KB activation of Nrf2 pathway.

## 5. Conclusions

**Figure 6:**
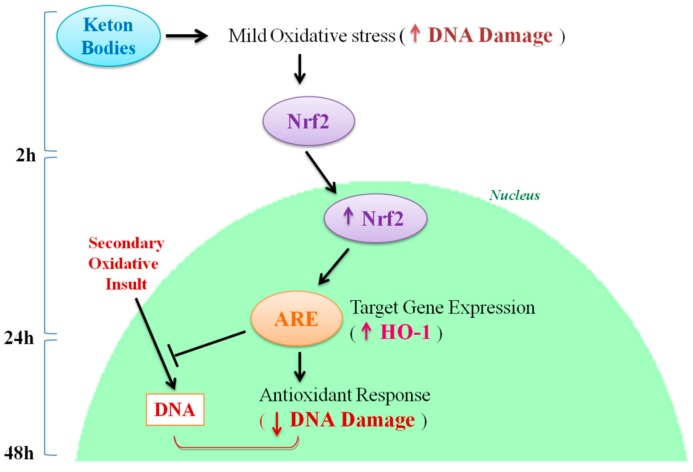
Proposed mechanism of action of KB in HMEC-1 cells.

Our results demonstrate the activation of a metabolic response in the cell due to effect of KB exposure. KB induce moderate oxidative stress, which activates the transcription factor Nrf2. Indeed, Nrf2 is stabilized and translocates into the nucleus. Here, Nrf2 binds to the ARE and activates the transcription of target genes, including HO-1. Consequently, the metabolic response caused by KB exposure makes cells more resistant to a secondary insult, in this case H_2_O_2_, leading to a reduction in DNA oxidative damage (Figure 6).

The novelty of our paper is that the effects of KB were investigated in endothelial cells, somewhat distant from the neurological context. It is important to underline that KB, and more generally a KD, could have an impact on cell metabolism in different tissue functions. The beneficial effects of a KD applied as a therapy have been clearly demonstrated [1,2,5,7], but the use of this diet as a weight loss method is still controversial. The mechanism hypothesized here is that KB could activate the transcription factor Nrf2, which enhances the cellular capacity to detoxify and eliminate harmful substances by the activation of cellular defense processes [20,21,22]. Actually, it is worth noting that the up-regulation of Nrf2 does not always lead to protection; for example, it may promote carcinogenesis in certain tissues. This could happen due to the other activities of Nrf2, such as suppression of apoptosis, improvement of mitochondrial function and redirection of glucose metabolism toward NADPH generation and anabolic pathways, which overall lead to cell proliferation [23,54,61,62]. Finally, it should be considered that the activation of Nrf2 is the result of impaired oxidative cell status, and could be harmful in the long term. A careful assessment of the risk-benefit ratio should be done every time that a KD is applied, for therapeutic purposes or not.

## Figures and Tables

**Table 1:** List of primer used for real-time reverse transcription-polymerase chain reaction (RT-PCR).

Genes	Primer Sequences	
Nrf2	F: 5′-AGCACATCCAGTCAGAAACC-3′	R: 5′-TGAAACGTAGCCGAAGAAAC-3′
HO-1	F: 5′-CAACATCCAGCTCTTTGAGG-3′	R: 5′-AGAAAGCTGAGTGTAAGGAC-3′
GAPDH	F: 5’-AGGGCTGCTTTTAACTCTGG-3′	R: 5′-CATGGGTGGAATCATATTGG-3′
